# Increased levels of pentraxins protein and cytokines bear good association in patients with severe dengue infection

**DOI:** 10.1038/s41598-020-80144-0

**Published:** 2021-01-12

**Authors:** Goutam Patra, Bibhuti Saha, Sumi Mukhopadhyay

**Affiliations:** 1 Department of Laboratory Medicine, Calcutta School of Tropical Medicine, Kolkata, West Bengal India; 2 Department of Tropical Medicine, Calcutta School of Tropical Medicine, Kolkata, West Bengal India

**Keywords:** Immunology, Biomarkers, Diseases, Health care, Medical research

## Abstract

Dengue is an arboviral infection with high rates of morbidity and mortality throughout the tropics and sub-tropics. This work studied the status of pentraxin (CRP/SAP) protein, ferritin, TNF-α and IL-1β levels in Dengue patients of different pathophysiological manifestations. Accordingly, clinically confirmed Dengue cases (n = 97) were enrolled and subsequently blood parameters were studied by Haematology cell counter and Biochemistry Autoanalyser. CRP, SAP, ferritin, TNF-α and IL-1β ELISA were done in all the samples by using standard ELISA kits. Statistical Analysis was done in all the experiments. The levels of CRP (*p* < 0.0001), SAP (*p* < 0.0001), ferritin (*p* < 0.0001), TNF-α (*p* < 0.0001) and IL-1β (*p* < 0.0001) were high in patients with Severe Dengue as compared to Dengue without warning signs. High levels of SGOT, SGPT and decreased platelet counts were found in severe patients as compared to Healthy donor. CRP/SAP as well as TNF-α/IL-1β were independently associated with both dengue severity and overall disease manifestation. Statistically significant increased CRP, SAP, ferritin, TNF-α and IL-1β titres were correlated in patients with severe clinical manifestations as compared to mild disease forms of dengue. Elevated levels of pentraxin, TNF-α/IL-1β in blood during dengue infection could act as an early predictor in Severe Dengue infection.

## Introduction

Dengue infection has become a serious problem in many countries, with a dramatic increase worldwide. It is estimated that more than 20,000 people die for dengue each year^1^. Severe dengue is the most dangerous form of dengue infection. Severe Dengue is characterized by a severe plasma leak resulting in shock, fluid accumulation with respiratory distress, severe bleeding assessed by the physician, severe damage to the organs of the liver, kidney, heart and other organs. Some patients have dengue fever without warning signs (DwoWS) and approximately 20 to 30% of them have dengue fever with warning signs (DwWS). During the last outbreak in West Bengal, a significant number of people were affected by significant morbidity and mortality. In addition to the usual hemorrhagic manifestations, significant morbidity has been observed in terms of organ involvement (hepatic, renal, cardiac, neurological manifestations, etc.). Large number of patients have experienced gastrointestinal manifestations. More recently, Mandal et al., from the Calcutta Medical College, observed atypical characteristics such as transaminitis and various neurological manifestations present in 83.83% and 11.11% cases, respectively. So far, there is no adequate treatment, an antiviral drug, although some prospective solutions are currently being studied among different groups of scientists^2^. Early management, vector control and socio-educational programs are the only methods to reduce the burden of morbidity and mortality worldwide^3^.

Currently, several tests can confirm dengue infection at the point of service. However, none of these tests can significantly predict the severity of the symptoms of the disease. During the onset of fever, it is difficult to distinguish between severe dengue fever and non-severe dengue fever. Since early hospitalization and supportive care can minify the case mortality rate of severe dengue fever, it is convenient to quickly identify patients who may develop severe dengue fever. Therefore, it is important to understand the immune pathogenesis of dengue infection to detect severe dengue fever at the beginning of infection. The pathogenesis of dengue infection is related to many factors, including the vector, the host genetic factor and the immune response^4–7^.

Colafrancesco et al. demonstrated that various acute-phase proteins play a crucial role to assess the asymptomatic inflammation. Acute-phase proteins (APP) are produced mainly by hepatic cells in response to various antigen-presenting cell-mediated cytokines, mostly IL-1β and IL-6. Previously our study also demonstrated that a high level of IL6 and vasodilation agent vascular endothelial growth factor (VEGF) were noted in severe dengue patients^8,9^. In this study, we also investigate the status of another important vasodilating factor TNF-α. Interestingly IL-1β also involves the up-regulation of the pleiotropic cytokine IL-6, which is a key element in priming hepatic cells for APP production^10,11^. Further, TNF-α and IL-1β collectively/interdependently orchestrate the liberation of different chemokines and boosting the rapid and prompt recruitment of various leukocytes towards the inflammatory area. Hofmann et al., also demonstrated that TNF-α is also responsible for inflammation by inducing local vasodilation and vascular leakage in various infections.

Short pentraxin protein, such as C-reactive protein (CRP), Serum amyloid P are the oldest proteins that are positively regulated in a disease state in a very short time. CRP and SAP are synthesized by the liver during benign infections. An almost 1000-fold increase in CRP levels was recorded within four hours after the onset of the inflammatory disease like bacterial infection^12^. Previously, CRP had been reported as a beneficial biomarker in colorectal cancer^13^, rheumatoid arthritis^14,15^, coronary heart disease (CH)^16^ and chronic kidney disease (CRM) at the beginning of bacterial infection^17^, viral infection^18^, HIV infection^19^, parasitic infection of malaria^20^. In addition, Mairuhu et al., 2005 demonstrated the overexpression of pentraxin 3 in patients with severe dengue, but pentraxin 2 mainly SAP was not studied^21^. Subsequently, Ray et al. 2012^22^ reported high SAP expression in patients with DF; However, its differential expression in patients with DWWS/SD (if any) remains unexplored. In addition, a strong expression of CRP in different degrees of dengue has also been reported^21^.

The serum amyloid P component, a pentameric glycoprotein, is also known as a diagnostic bio-marker for amyloidosis^23^ and Alzheimer's disease^24^. SAP proteins play a crucial role in bacterial infections^25^, viral infections^26^ and atherosclerosis^27^. As IgM antibodies against dengue may not be detectable until the fifth to eighth day of the disease and NS1 disappears from the blood much earlier in a secondary infection due to the presence of neutralizing antibodies, diagnosis becomes challenging. Taken together, in search of a biological marker of severity, we studied for the first time the status of inflammatory biomarkers such as CRP and SAP in patients with primary and secondary dengue infection.

## Result

### Demography and clinical parameters

In this study, 2772 patients suspected of having dengue fever were hospitalized at the OPD or were hospitalized at the beginning of infection at Carmicheal Hospital of Tropical Diseases, Kolkata, West Bengal, in India. Out of 2772 patients, 97 (3.49%) were positive for NS1/IgM/IgG anti-Dengue antibody. Twenty healthy subjects and 20 other febrile infection like malaria, chikungunya served as study control and were designated HD and OFI respectively. On the basis of clinical manifestations, 26 were classified as DWWS and 9 patients with SD. Classification was based on clinical history and clinical characteristics of selected patients, followed by WHO guidelines. Among the patients infected with dengue, approximately 43.29% (n = 42) were men and 56.69% (n = 55) were women. Out of the 97 patients with confirmed dengue fever, 41 patients were classified as secondary dengue (i.e., DwoWS (n = 27), DwWS (n = 11), and SD (n = 3)) (Table 1).

**Table 1:** Demography of study subjects.

Characteristics	No. of study subjects		
	DwoWS (%)	DWWS (%)	SD (%)
Total number of population	62	26	9
Male (number, percentage)	28 (45%)	10 (38%)	4 (44%)
Female (number, percentage)	34 (55%)	16 (62%)	5 (56%)
Primary dengue (number, percentage)	35 (56%)	15 (58%)	6 (66%)
Secondary dengue (number, percentage)	27 (44%)	11 (42%)	3 (34%)
Age (year, median)	31.62 (27)	32.33 (32)	31.62 (27)
Hospitalization (number, percentage)	29 (47%)	19 (73%)	9 (100%)
Positive dengue IgM test (number, percentage)	62 (100%)	26 (100%)	9 (100%)
Day of fever on enrollment (mean, median)	3.51(3)	3.56 (4)	3.55 (3)

All patients observed (respectively DwoWS, DwWS and SD) had fever, myalgia (39%, 85% and 100%), rash (60%, 65% and 77%), headache (71%, 81% and 88%)), vomiting (13%, 46% and 77%) and abdominal pain (34%, 61% and 77%) (Table S1).

### Laboratory parameters

**Figure 1:**
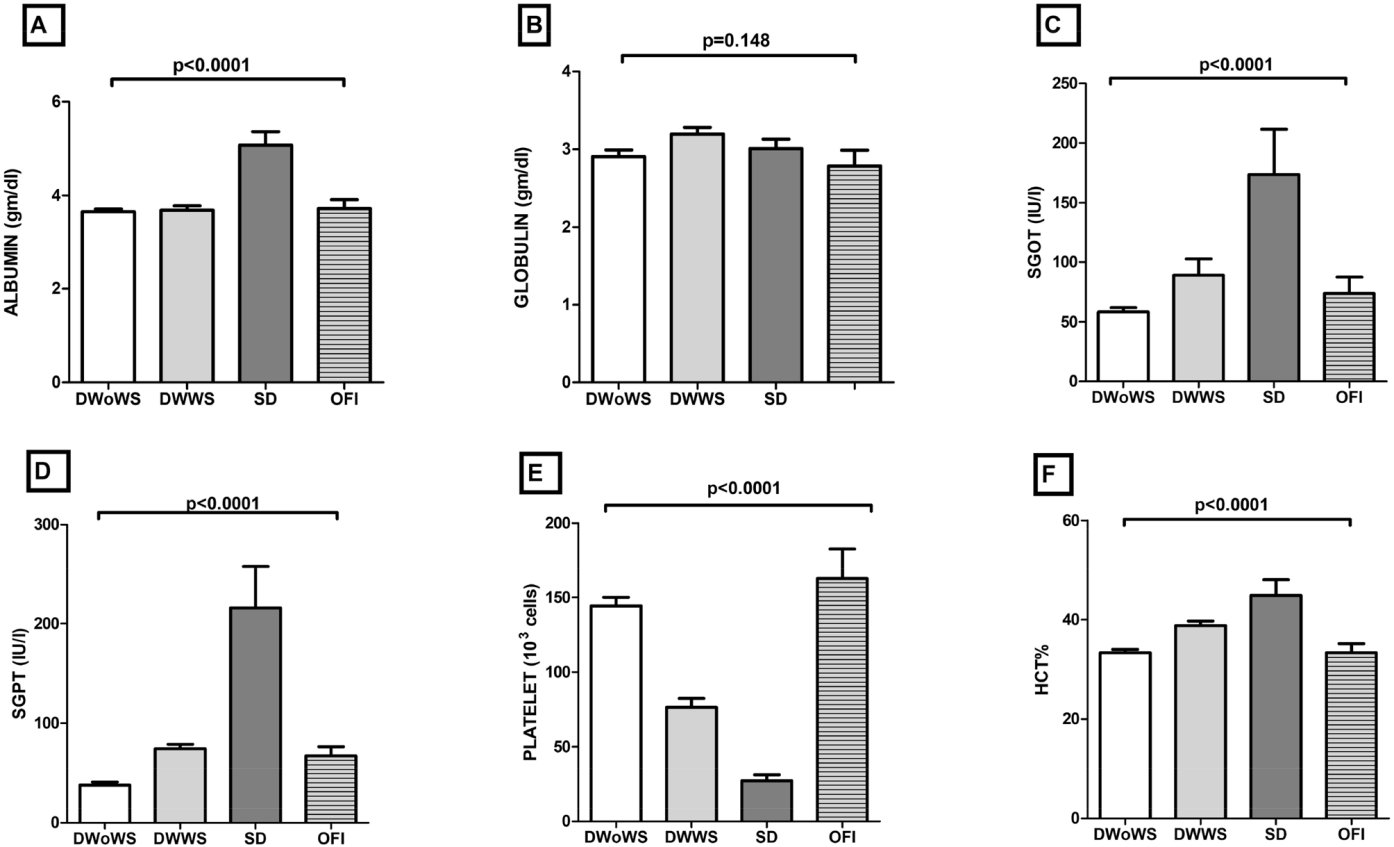
Level of serum (**A**) Albumin, (**B**) Globulin, (**C**) SGOT, (**D**) SGPT, (**E**) Platelet and (**F**) HCT of Dengue Without Warning Signs (DWoWS), Dengue With Warning Signs (DWWS), Severe Dengue (SD) versus Other febrile infection (OFI). Study subjects are significantly different from each category is indicated by *P* < 0.05. Statistical analysis was performed using the Graph-Pad Prism statistics software (Graph-Pad Software Inc., San Diego, CA, USA free demo version 5.04).

Seventh day of biochemical results in dengue patients showed that the low concentrations (median and IQR value) of SGOT (56 IU/L; IQR 34–86.25 IU/L) and SGPT (29 UI/L; IQR, 22–51 IU/L) were detected in the sera of patients with DwoWS. In patients with warning signs of the disease, SGOT levels (66 IU/L; IQR 34.25–128.3 IU/L) and SGPT (70 IU/L; IQR 57.57–90.50 IU/L) were lower than those of patients with SD (154.50 IU/L; IQR 69–278 IU/L and 193 IU/L; IQR 100.5–340.5 IU/L respectively). SD patients had decreased thrombocyte counts approximately fourfold and 2.18-fold, respectively as compared to DwoWS and DWWS. In addition, ANOVA analysis between DwoWS, DWWS and SD with respect to SGPT (*p* < 0.0001), platelets (*p* < 0.0001), globulin (*p* = 0.148) and hematocrit (*p* < 0.0001) showed a significant difference (Fig. 1). However, the day 3 biochemical results of dengue infection in severe patients did not show statistically different results as compared to the healthy donor except, CRP (*p* = 0.0006), SAP(*p* < 0.0001), ferritin (*p* = 0.0004), biochemical parameter SGOT (*p* = 0.024), SGPT (*p* = 0.029) and platelets (*p* = 0.007) (Figure S1).

### Quantification of acute phase protein

**Figure 2:**
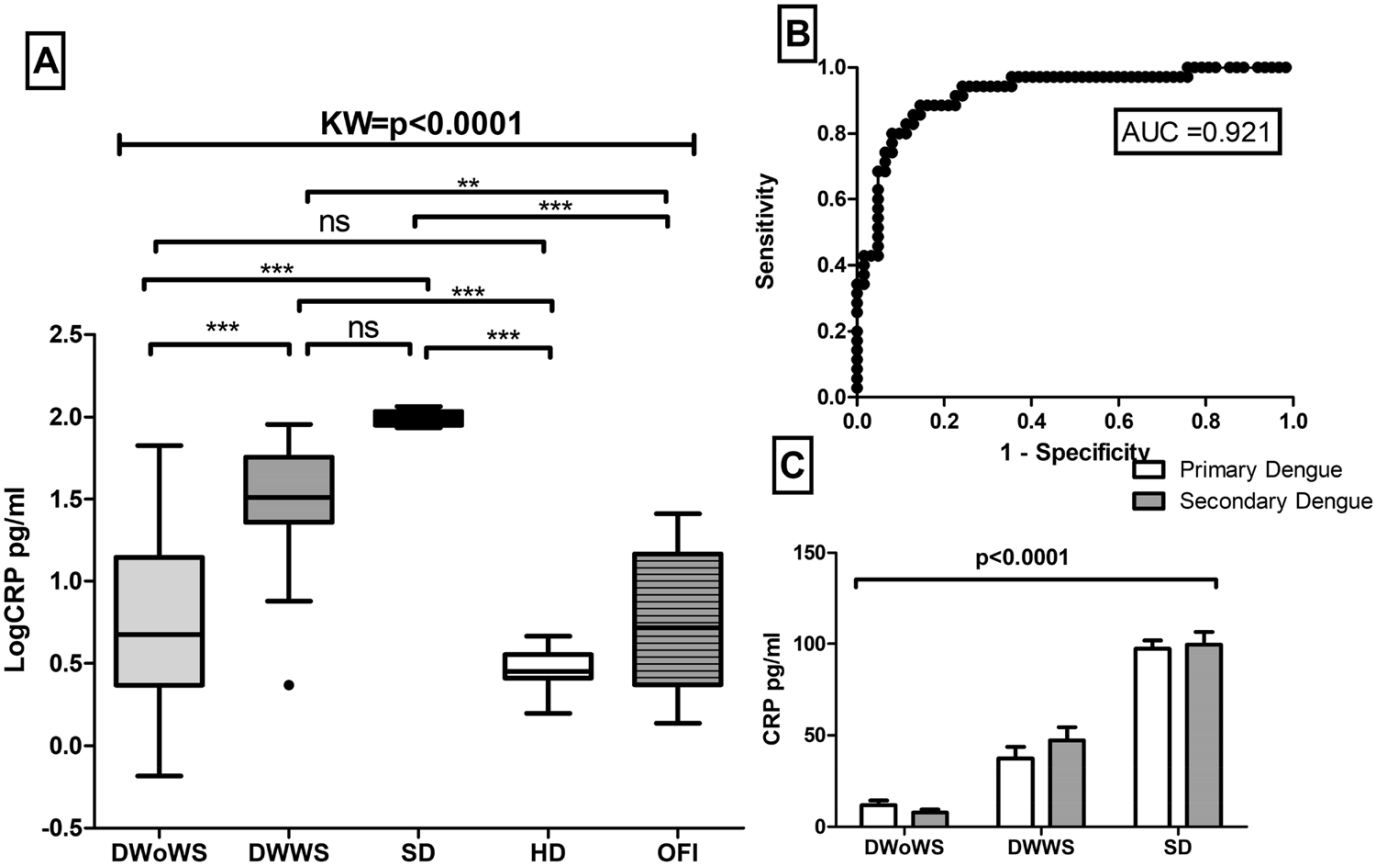
(**A**) Levels of C Reactive Protein (CRP) of dengue without warning sign (DWoWS), dengue with warning signs (DWWS), severe dengue (SD) and Other febrile infection (OFI). Study subjects are significantly different from each category. (**B**) Receiver operating characteristics curve analysis obtained the AUC = 0.921. (**C**), Levels of CRP of primary and secondary dengue infection. Study subjects are significantly different from each category is indicated by *P* < 0.05. Statistical analysis was performed using the Graph-Pad Prism statistics software (Graph-Pad Software Inc., San Diego, CA, USA free demo version 5.04).

**Figure 3:**
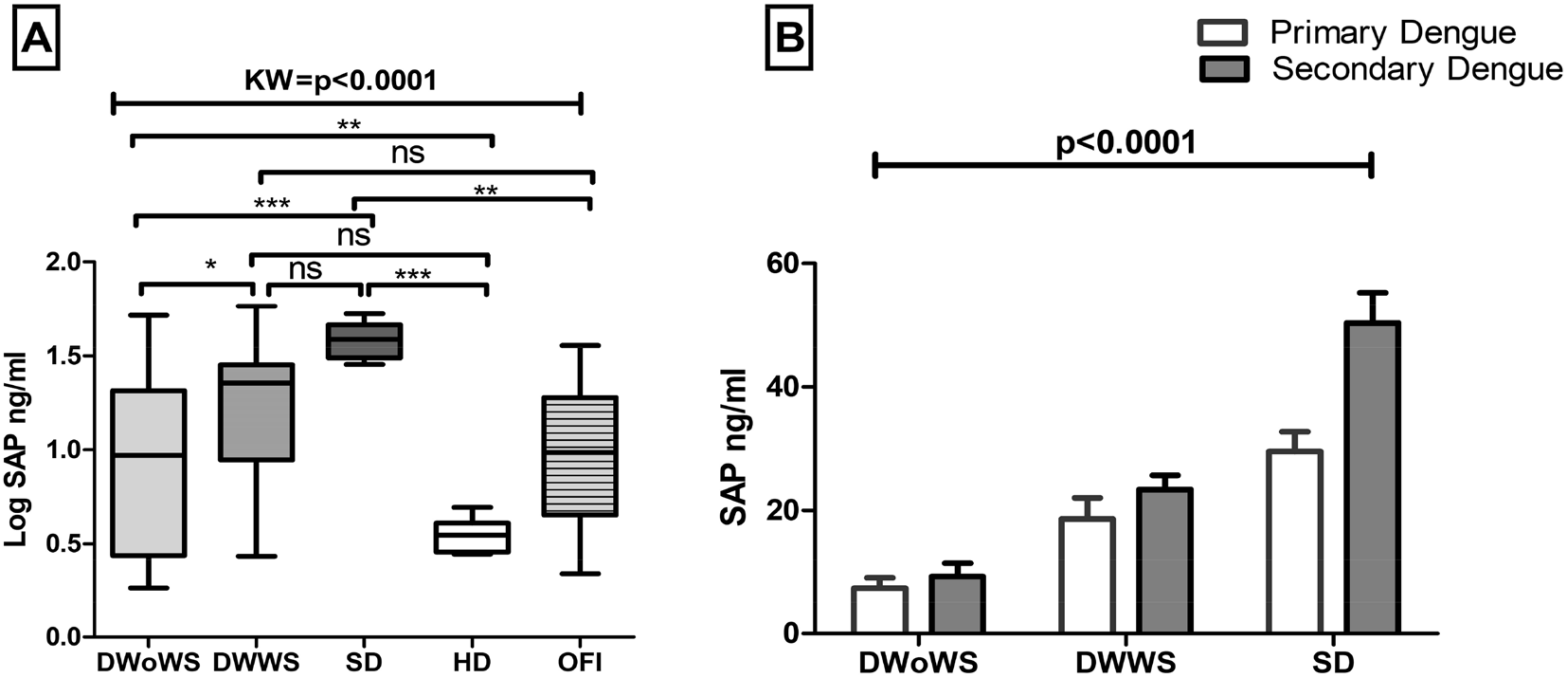
(**A**) Levels of Serum Amyloid P (SAP) protein of dengue without warning sign (DWoWS), dengue with warning signs (DWWS) severe dengue (SD) and Other febrile infection (OFI). Study subjects are significantly different from each category. (**B**) Levels of SAP of primary and secondary dengue infection. Study subjects are significantly different from each category is indicated by *P* < 0.05. Statistical analysis was performed using the Graph-Pad Prism statistics software (Graph-Pad Software Inc., San Diego, CA, USA free demo version 5.04).

**Figure 4:**
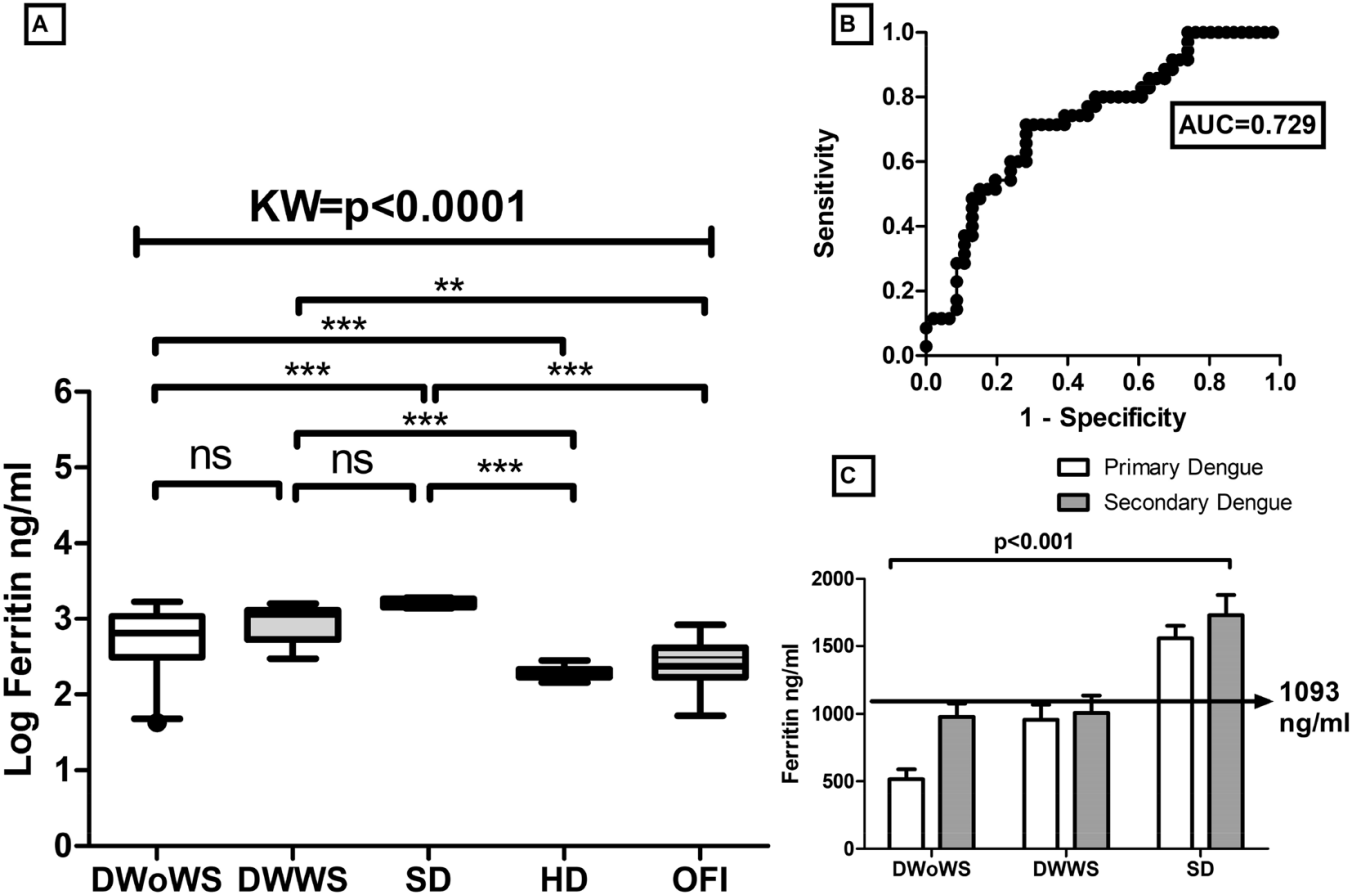
(**A**) Levels of Ferritin protein of dengue without warning sign (DWoWS), dengue with warning signs (DWWS), severe dengue (SD) and Other febrile infection (OFI). Study subjects are significantly different from each category. (**B**) Receiver operating characteristics curve analysis obtained the AUC = 0.729. (**C**) Levels of Ferritin of primary and secondary dengue infection. Arrow line showing the severity cut‐off value (1093 ng/mL) obtained from the Youden index. Study subjects are significantly different from each category is indicated by *P* < 0.05. AUC, area under the curve; HD, healthy donors.Statistical analysis was performed using the Graph-Pad Prism statistics software (Graph-Pad Software Inc., San Diego, CA, USA free demo version 5.04).

Initially, circulating levels of CRP were evaluated in different patients with dengue, i.e. DwoWS (median 4.736 pg/ml, IQR 2.337–14.07 pg/ml), DWWS (median 32.40 pg/ ml, IQR 22.90–57.04 pg/ml) and SD (median 96.15 pg/ml, IQR 89.23–108.2 pg/ml) ml) compared to HD (median of 2.82 pg/ml, IQR 2.57–3.58 pg/ml) were significantly higher in patients with severe dengue. However, CRP levels in OFI were very low (median 5.22 pg/ml, IQR 2.35–14.28 pg/ml) as compared to SD (*p* < 0.0001) and DWWS (*p* < 0.001) (Fig. 2). In addition, another acute phase protein, SAP was also significantly increased in severe dengue infection (median of 38.43 ng/ml, IQR 30.97–46.48 ng/ml) versus HD (median value 3.51 ng/ml, IQR 2.85–4.1 ng/ml). However, SAP was not significantly increased in DWoWS as compared to DWWS. SAP levels in OFI were low as compared to SD (*p* < 0.001) (Fig. 3). Further another acute phase protein ferritin levels also increased in SD (1628 ng/ml, IQR 1430–1852 ng/ml) as compared to DWWS (1160 ng/ml, IQR 530–1307 ng/ml). However, ferritin levels in OFI were very low (238.1 ng/ml, IQR 170.5–420.6 ng/ml) as compared to SD (*p* < 0.0001) and DWWS (*p* < 0.001) (Fig. 4). On the other hand, 3.81 fold and 9.09 fold increased ferritin levels was also observed in DWoWS and SD patients as compared to HD (Fig. 4). In addition, a dengue severity threshold was applied to differentiate severe dengue fever from the less severe forms of this disease. These data show the severity threshold value for ferritin is 1093 ng/ml, (sensitivity 71% and specificity 72%) as compared to the less severe forms of dengue fever (Fig. 4C). The levels of acute phase proteins were also analyzed in context to primary versus secondary infections (Figs. 2C, 3B, 4C). Higher levels of CRP, SAP and Ferritin were observed in secondary cases compared to primary dengue cases which was statistically significant (*p* < 0.0001).

### Quantification of cytokines

**Figure 5:**
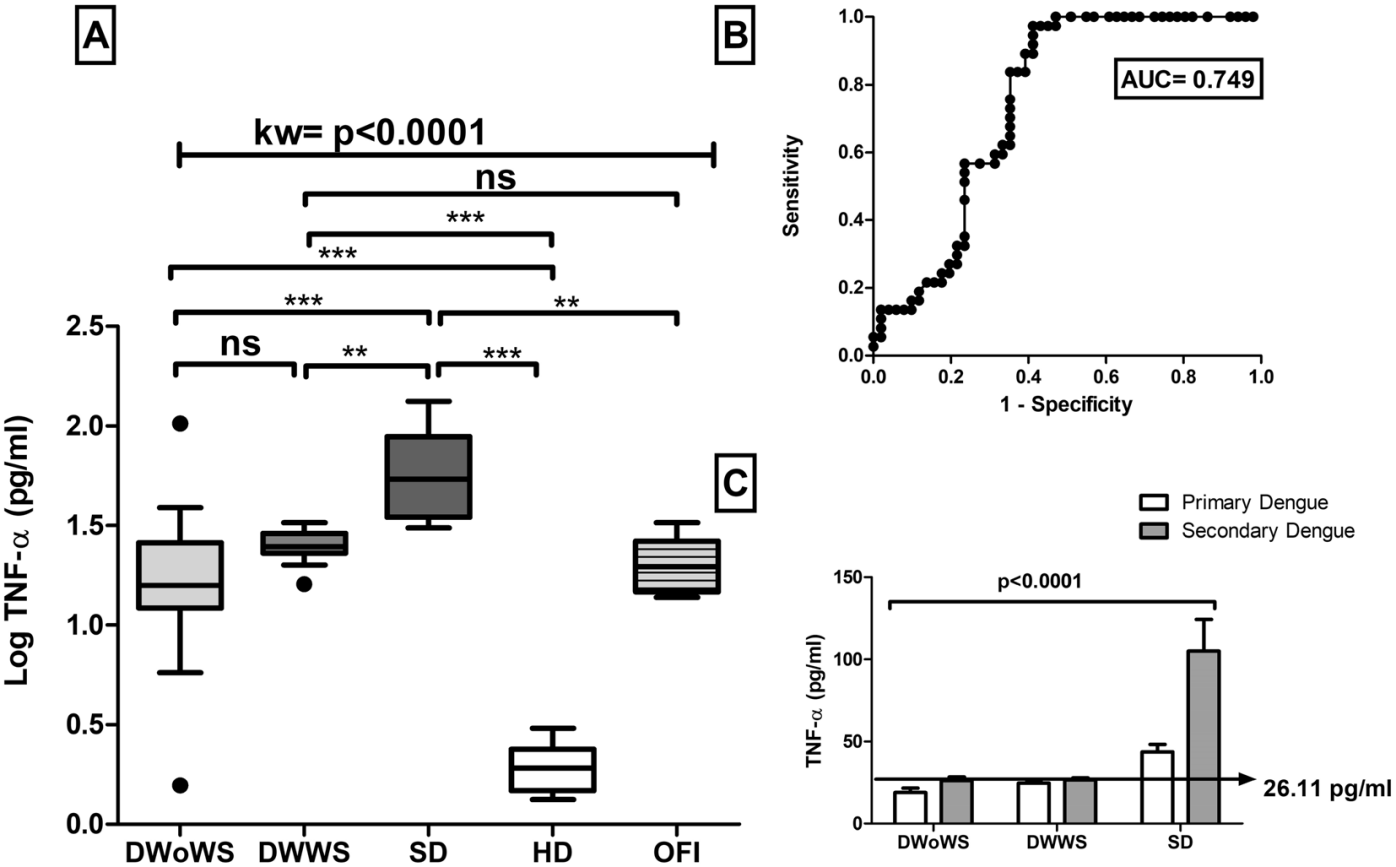
(**A**) Levels of TNF-α of dengue without warning sign (DWoWS), dengue with warning signs (DWWS), severe dengue (SD) and Other febrile infection (OFI). Study subjects are significantly different from each category. (**B**) Receiver operating characteristics curve analysis obtained the AUC = 0.749. (**C**) Levels of TNF-α of primary and secondary dengue infection. Arrow line showing the severity cut‐off value (26.11 pg/mL) obtained from the Youden index. Study subjects are significantly different from each category is indicated by *P* < 0.05. AUC, area under the curve; HD, healthy donors. Statistical analysis was performed using the Graph-Pad Prism statistics software (Graph-Pad Software Inc., San Diego, CA, USA free demo version 5.04).

**Figure 6:**
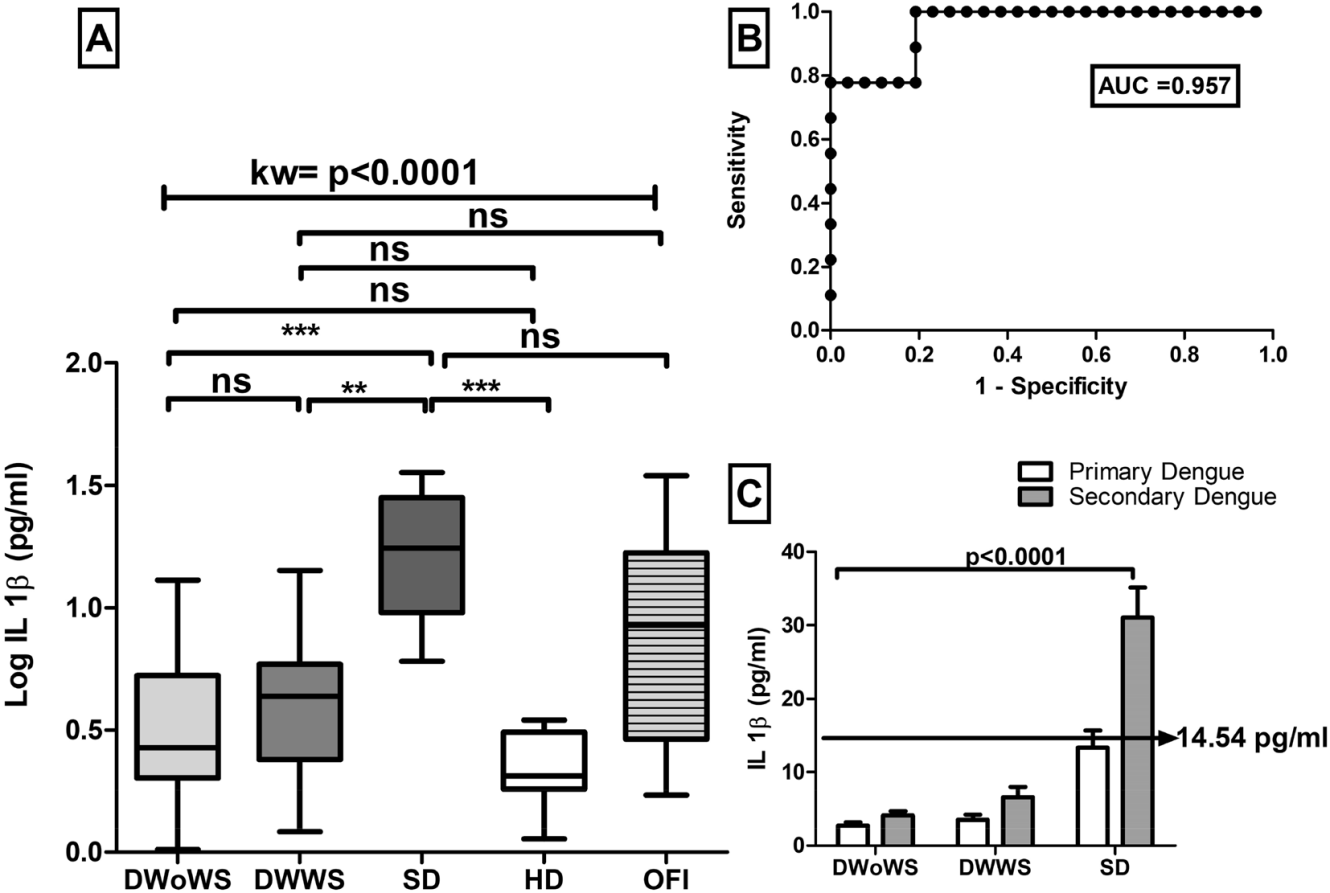
(**A**) Levels of IL-1β of dengue without warning sign (DWoWS), dengue with warning signs (DWWS), severe dengue (SD) and Other febrile infection (OFI). Study subjects are significantly different from each category. (**B**) Receiver operating characteristics curve analysis obtained the AUC = 0.957. (**C**) Levels of IL-1β of primary and secondary dengue infection. Arrow line showing the severity cut‐off value (14.54 pg/mL) obtained from the Youden index. Study subjects are significantly different from each category is indicated by *P* < 0.05. AUC, area under the curve; HD, healthy donors. Statistical analysis was performed using the Graph-Pad Prism statistics software (Graph-Pad Software Inc., San Diego, CA, USA free demo version 5.04).

During the febrile phase of infection significant levels of the circulating cytokines were elevated in different patients with dengue. Previously our lab demonstrated that high levels of IL6 were observed in severe dengue patients as compared to HD (Patra et al. 2019). Now in this study we also observed high levels of TNF-α in SD (53.48 pg/ml, IQR 32.1–91.26 pg/ml), DWWS (24.86 pg/ml, IQR 23.3–28.84 pg/ml) and DWoWS (15.85 pg/ml, IQR 12.16–25.98 pg/ml) as compared to HD (1.91 pg/ml, IQR 1.47–2.38 pg/ml). Hence, 3.37 fold increased TNF-α was observed in SD patients as compared to DWoWS. However,TNF-α levels in OFI were very low as compared to SD (*p* < 0.001 (Fig. 5). In addition, leukocyte endogenous mediator IL-1β was also significantly increased in severe dengue infection (median of 17.52 pg/ml, IQR 10.51–28.71 pg/ml) versus HD (median value 2.00 pg/ml, IQR 1.09–2.88 pg/ml). However, IL-1β was not significantly increased in DWoWS as compared to DWWS (Fig. 6). Hence a dengue severity threshold was applied to differentiate severe dengue fever from the less severe forms of this disease. These data show the severity threshold value for TNF-α (26.11 pg/ml) and IL-1β (14.54 pg/ml) respectively, as compared to the less severe forms of dengue (Figs. 5C and 6C).

The levels of circulating cytokines were also analyzed in context to primary versus secondary infections (Figs. 5C and 6C). Higher levels of TNF-α and IL-1β were observed in secondary dengue infections compared to primary dengue cases which was statistically significant (*p* < 0.0001).

### Liver and blood-related changes in association with acute phase proteins and serum cytokines in circulating levels

**Figure 7:**
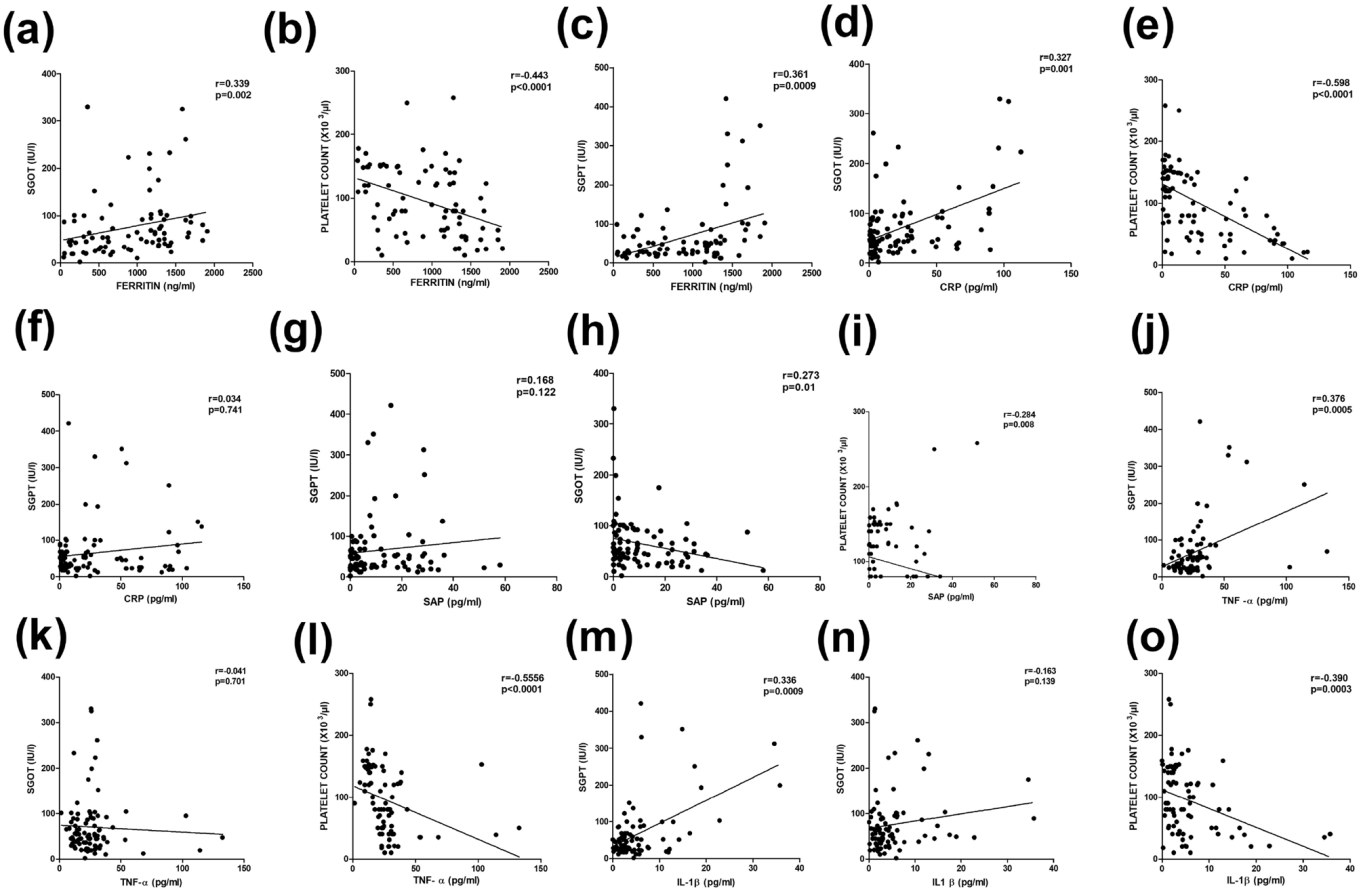
Correlation of (**a**) SGOT versus Ferritin, (**b**) Platelet versus Ferritin, (**c**) SGPT versus Ferritin, (**d**) SGOT versus CRP, (**e**) Platelet versus CRP, (**f**) SGPT versus CRP, (**g**) SGPT versus SAP, (**h**) SGOT versus SAP, (**i**) Platelet versus SAP (**j**) SGPT versus TNF-α, (**k**) SGOT versus TNF-α, (**l**) Platelet versus TNF-α, (**m**) SGPT versus IL-1β, (**n**) SGPT versus IL-1β and (**o**) Platelet versus IL-1β in patients with dengue infection. Statistical analysis was performed using the Graph-Pad Prism statistics software (Graph-Pad Software Inc., San Diego, CA, USA free demo version 5.04).

Changes in liver function are commonly described in severe dengue fever (Nguyen et al. 2004). Out of the 9 patients with SD infection, 77% suffered from whole abdominal pain and liver problem in this study. These patients had higher levels of CRP and SAP compared to those for whom symptoms were absent. The increase in SGOT levels was positively correlated with CRP (r = 0.327 with *p* = 0.001), SAP (r = 0.273 with *p* = 0.01), while the platelet was negatively correlated with CRP (r =  − 0.598 with *p* < 0.0001). Interestingly, the platelet was found to negatively correlate with SAP levels (r =  − 0.0284 with *p* = 0.008). These data indicate a possible influence of these acute phase proteins on elevation of SGOT and painful liver enlargement. Further, serum cytokine levels TNF-α was positively (r = 0.376 with *p* = 0.0005) and negatively (r =  − 0.556 with *p* < 0.0001) correlated with SGPT and platelet respectively. On other hand, elevated liver SGPT levels was positively correlated with IL-1β (r = 0.363 with *p* = 0.0009), while the platelet was inversely correlated with IL-1β (r =  − 0.390 with *p* = 0.0003). These association indicate that IL-1β and TNF-α probably induces liver tissue injury, vascular damage and thrombocytopenia (Fig. 7).

## Discussion

Dengue fever is an unpredictable viral disease. Early identification and management of severe dengue patients are very crucial step to prevent death rate until the discovery of anti dengue drug. To our knowledge, no specific study has been conducted to date on the pentraxin profile in primary and secondary dengue fever in Kolkata, a hyperendemic dengue zone in eastern India. In this study, we studied the status of inflammatory proteins in the acute phase in patients with dengue. Many host as well as pathogen related factors contribute to the pathogenesis of the dengue virus. Several components are produced from hepatic cells infected with the dengue virus, such as CRP, SAP and ferritin in the early stage of infection. The first described acute phase protein (APP) was C-reactive protein (CRP), a positive APP identified in humans^28^. CRP generally binds to pathogens, reinforcing their opsonization, before the production of specific immunoglobulin (IgM and IgG). CRP belongs to the family of pentraxin proteins and is used as a biomarker of inflammation, especially for bacterial infections^29^. In general, it is assumed that viral infection increases CRP levels by 10 to 40 mg/L^29–31^. In our study, the median CRP level was significantly elevated in people with severe dengue compared with the less severe form (DwoWS/DWWS). The critical phase of dengue occurs more frequently towards the end of the febrile stage, usually after the third day of the disease^32^. In particular, we found a significant association of higher levels of CRP with severe dengue fever during the first 3 days of the disease (febrile phase). Previously, Mairuhu et al., 2005, had shown that the level of CRP was very high among people with severe dengue compared to non-severe dengue in the population of the Netherlands^21^. However, the status of CRP in severe secondary dengue remains unexplored in the population of eastern India. Pentraxin protein SAP have also not been studied. Subsequently, Ray et al. 2012^22^ reported high SAP expression in patients with SD; however, its differential expression in patients with DWWS/SD (if present) remains unexplored. In addition, high levels of SAP have been reported in dengue infection, which play an important role in the opsonized pathogen or in other cells that are dead due to their phagocytic clearance^33^. It is interesting to note that in this study, significantly elevated levels of pentraxin proteins were observed in SD compared to DWWS (Figs. 3 and 4). On the other hand another serum APP ferritin levels also increased in SD patients as compared to HD and DWWS (Fig. 5). Serum APPs have also been associated with severe pathogenesis of dengue and strongly correlate with thrombocytopenia and high liver enzyme^33^. Our analysis showed that ferritin titre of 1093 ng/ml had 71% sensitivity for SD prediction during early phase of dengue fever (febrile phase). As the vascular permeability, bleeding, ascitis are the main symptoms of SD patients, we evaluated the levels of IL-1β in dengue patients at the early stage of infection. Here we found very high levels of serum IL-1β in SD patients as compared to DWWS (*p* < 0.001) however the levels of IL-1β in DWoWS were negative as compared to HD. This data suggested that high levels of IL-1β in SD patients could play crucial role in its pathogenesis. Previously JR Bethea et al., 1992 demonstrated that IL-1β induced the production of TNF-α, from the monocyte^34^. TNF-α is mufti-functional cytokine that could affect on endothelial cells^35^ and take part in certain manifestations of DWWS and SD, such as low platelet count, internal or external hemorrhage, ascitis etc.^36^ Although DWoWS patients were negative for TNF-α, but elevated levels have been observed in DWWS and SD compared to that of healthy donor (*p* < 0.0001). These data support that TNF-α production in SD and DWWS patients is connected to immuno-pathology of severe dengue infection. To our knowledge, the comparative study of serum pentraxin (SAP/CRP) protein, ferritin, TNF-α and IL-1β levels in patients with severe secondary and primary dengue fever in eastern India has not yet been well studied and our current study will help to initiate more studies to unravel the potential of pentraxin protein as a biomarker in dengue severity.

Taken together, all these acute phase proteins and TNF-α/IL-1β increase the severity of the disease. Given the important role played by APP and cytokines in the pathogenesis induced by viruses, its association with different biochemical parameters was also studied. All clinical manifestations, joint pain, retro-orbital pain, headache and muscle pain were common to all patients with dengue fever. On the other hand, it has been shown that vomiting, abdominal pain and organ damage are positively associated with SD and could be used as prognostic symptoms of SD. Organ involvement, specially liver has been well documented in severe form of dengue infection. In addition, in our study, biochemical parameters such as high liver enzyme like SGPT, SGOT, albumin, globulin, haematocrit and low thrombocyte count were well documented in patients with SD as compared with DwoWS. However, a slight statistically significant difference was ascertained in the routine haematological as well as biochemical test between patient with severe dengue and HD only for SGPT, SGOT, platelets and APP/cytokines on day 3 of the infection. Therefore, according to biochemical parameters, it was difficult to distinguish SD from DwoWS in the early stages of infection. Therefore, we quantified and established a specific threshold value for the APP and cytokines that could provide us with a clear indication of the severity of dengue fever. Therefore, our research revealed that among the APP analyzed, based on the specific cut-off value of CRP, ferritin, TNF-α and IL-1β it could act as a severity biomarker. Inflammatory APP, such as increased CRP/SAP, has also been significantly associated with decreased platelet counts and increased SGPT in patients with severe dengue. Hepatomegaly in patients with severe dengue was well documented, which was reflected again in an increase in SGOT and serum APP. Similarly TNF-α and IL-1β was also associated with platelet destruction and hepatic cell injury. Our data revealed that most severely ill patients have accumulation of fluid in the liver and lungs; Decrease in the number of platelets, increase in APP titres. The two APPs were found to have slightly higher titers in secondary infections than in primary infections. The data from this study reveal that the increase in pentraxin proteins are well related to the severity of dengue fever in the first days of infection. The study limitation is the evaluation of these data in a much larger patient population should be used before a global clinical application.

## Materials and methods

### Declaration of ethics

The Clinical Research Ethics Committee, Calcutta School of Tropical Medicine, West Bengal, Kolkata, India, on Human Ethics approved the study (Ethical Number - CREC-STM/275 of 18.04.2015) and took the necessary blood samples from the healthy control and patients. A written informed consent was obtained from both enrolled patients and the healthy control population. All methods were performed in accordance with the relevant guidelines and regulations.

### Collection of blood samples

This was a hospital based control study. Dengue virus infected patients diagnosed at Calcutta School of Tropical Medicine (CSTM), West Bengal, India during August 2017 to September 2018 were enrolled. Patients with dengue-like disease (n = 2772) were examined and four milliliters (4.0 ml) of venous blood were collected by venipuncture and retained in EDTA anticoagulant tubes. Plasma was separated by centrifugation at 3000 rpm and subjected to NS1 MAC-ELISA, IgM/IgG ELISA (Pan Bio, East Brisbane, Australia). Out of the 2772 patients suspected, 97 NS1 and IgM/IgG were selected for our study, of which 62 were classified as dengue without warning signal (DwoWS) and 26 patients with the same type of dengue with warning signal (DWWS) and 9 others were severe (SD). In addition, 20 healthy donor (HD) and 20 other febrile infection (OFI) like malaria, chikungunya subjects were also enrolled for this study. Serum was collected for all subjects in the study and stored at − 20 °C until further use.

### Quantification of biochemical parameters

To characterize the pathological state of the study subjects, a detailed biochemical analysis of the blood samples was measured using a standard auto-analyzer (ERBA model No. EM360). These include serum glutamic oxaloacetic transaminase (SGOT), serum glutamic pyruvic transaminase (SGPT), globulin and albumin. In addition, white blood cell counts, red blood cell counts, hemoglobin (HB), hematocrit (HCT), and platelet count (PLT) were measured using an automatic cell counter. (model SYSMEX No -KX100). The tests were performed following the manufacturer's provided instructions. All samples were analyzed and the value was taken for analysis.

### Determination of acute phase protein titres in serum

To quantify acute serum protein levels in different groups of dengue infections, the stored sera were thawed and CRP, SAP and ferritin were evaluated using standard ELISA kits (Ray Biotech, GA). The ELISA tests were performed according to the manufacturer's instructions (Ray Biotech). The samples were analyzed and the median value was taken for analysis.

### Determination of serum cytokine titres in serum

To quantify inflammatory and anti-inflammatory serum cytokines levels in different groups of dengue infections, the stored sera were thawed and TNF-α and IL-1β were evaluated using standard ELISA kits (RayBiotech, GA). The ELISA tests were performed according to the manufacturer's instructions (Ray Biotech). The samples were analyzed and the median value was taken for analysis.

### Statistical analysis

Statistical analysis was performed using the Graph-Pad Prism statistics software (Graph-Pad Software Inc., San Diego, CA, USA free demo version 5.04) and all statistical methods were adopted from Patra et al., 2019^8,9^. Briefly, all data were subjected to ANOVA with post-hoc test for finding the significance of each study groups. Further, Mann–Whitney test was performed to estimate the statistical significance between each group. The correlation between APP/Cytokines and blood parameters was determined by using Spearman's correlation.

## Supplementary Information

Supplementary Information.

## Data Availability

The data of this study are available with SM upon reasonable request.
