# Supplementary material for: Increased levels of pentraxins protein and cytokines bear good association in patients with severe dengue infection
Source: Sci Rep. 2021 Jan 12;11:511. doi: 10.1038/s41598-020-80144-0 (PMC7804429; doi:10.1038/s41598-020-80144-0)
Supplement: Supplementary file 1 — Supplementary Information. [file 41598_2020_80144_MOESM1_ESM.docx]

**Supplementary Information**

**Increased levels of Pentraxins protein and cytokines bear good association in patients with severe dengue infection**

Goutam Patra^1^, Bibhuti Saha^2^ and Sumi. Mukhopadhyay^1*^

^1^Department of Laboratory Medicine, ^2^Department of Tropical Medicine Calcutta School of Tropical Medicine, Kolkata, India

*^*^****Corresponding author****: Dr.SumiMukhopadhyay, Department of Laboratory Medicine, Calcutta School of Tropical Medicine, West Bengal, India; Email:drsumimukhopadhyay@gmail.com*

**Table .S1. Clinical signs and symptoms of the study population**

| **Clinical signs and symptoms** | **DwoWS(%)**  **(n=62)** | **DWWS(%)**  **(n=26)** | **SD(%)**  **(n=9)** |
| --- | --- | --- | --- |
| **Rashes** | **37(60%)** | **17(65%)** | **7(77%)** |
| **Headache** | **44(71%)** | **21(81%)** | **8(88%)** |
| **Myalgia** | **24(39%)** | **22(85%)** | **9(100%)** |
| **Abdominal Pain** | **21(34%)** | **16(61%)** | **7(77%)** |
| **Vomiting** | **8(13%)** | **12(46%)** | **7(77%)** |
| **Loose motion** | **11(17%)** | **7(27%)** | **4(44%)** |
| **Bleeding** | **0** | **26(100%)** | **9(100%)** |
| **Thrombocytopenia** | **0** | **26(100%)** | **9(100%)** |


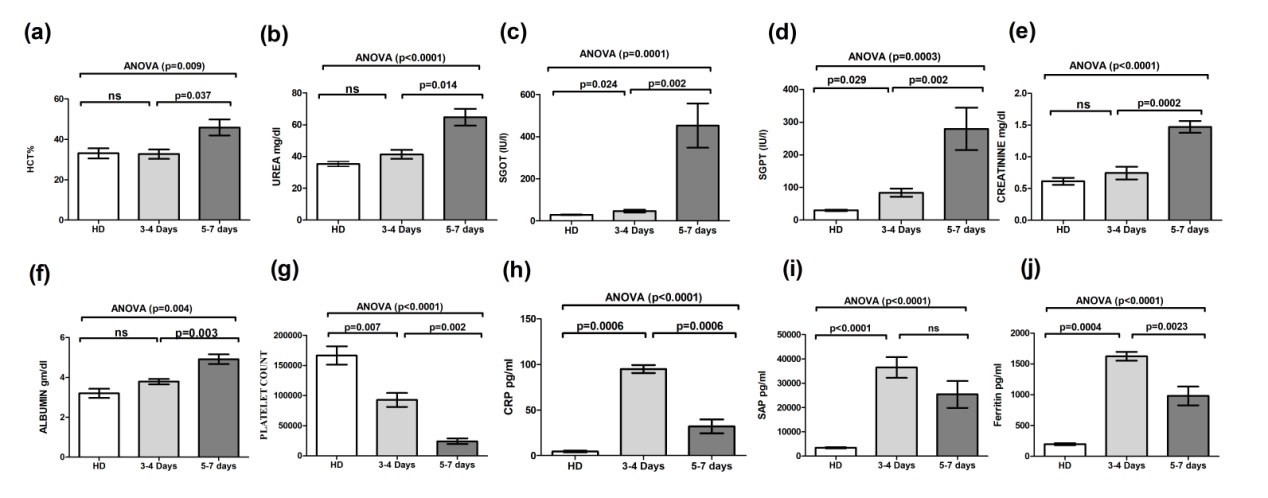


Figure S1: Level of serum (a) HCT, (b) Urea, (c) SGOT, (d)SGPT, (e) Creatinine , (f) Albumin, (g) Platelet, (h) CRP, (i) SAP and (j) Ferritin of Dengue Without Warning Signs (DWoWS), Dengue With Warning Signs (DWWS) vs. Severe Dengue (SD) in 3-4 days and 5-7 days of infections.Study subjects are significantly different from each category is indicated by *P*<0.05
